# A Comprehensive Evaluation of ZrC Nanoparticle in Combined Photothermal and Radiation Therapy for Treatment of Triple-Negative Breast Cancer

**DOI:** 10.3389/fonc.2021.801352

**Published:** 2021-12-21

**Authors:** Shan Jiang, Zhao Liu, Yuhang Tian, Ming Zhuang, Shiqi Piao, Yan Gao, Andrew Tam, Hongtao Hu, Wen Cheng

**Affiliations:** ^1^ Department of Ultrasound, Harbin Medical University Cancer Hospital, Harbin, China; ^2^ Department of Radiation Oncology, Harbin Medical University Cancer Hospital, Harbin, China; ^3^ School of Chemistry and Chemical Engineering, Harbin Institute of Technology, Harbin, China; ^4^ Department of Radiation Oncology, City of Hope National Medical Center, Duarte, CA, United States

**Keywords:** sensitizer, PTT, RT, nanoparticle, breast cancer

## Abstract

Because of the difficulty in treating triple-negative breast cancer (TNBC), the search for treatments has never stopped. Treatment opinions remain limited for triple-negative breast cancer (TNBC). The current treatment approach of using photothermal therapy (PTT) is often imprecise and has limited penetration below the surface of the skin. On the other hand, radiation therapy (RT) has its unavoidable disadvantages, such as side effects or ineffectiveness against hypoxic tumor microenvironment (TME). In this study, we proposed the use of ZrC nanoparticles in conjunction with RT/PTT to enhance antitumor and antimetastatic effect. We modified the ZrC nanoparticle with bovine serum albumin (BSA) and folic acid (FA), sizing desirable about 100nm. The photothermal conversion efficiency was calculated to be 40.51% and sensitizer enhancement ration (SER) was 1.8. With addition of ZrC NPs, more DNA were damaged in γ-H2AX and more ROS were detected with immunofluorescence. *In vitro* and vivo, the combined therapy with ZrC NPS showed the best effect of tumor cell inhibition and safety. Our results provide evidence that the combination of ZrC NPs, PT, and RT is effective in of TNBC, making it a great potential application for cancer therapy in clinic.

## Introduction

Radiation therapy (RT), combined with surgery and systemic chemotherapy, is the current standard of care treatment for breast cancer (1, 2). Using high energy radiation such as X-ray or γ-ray, RT directly ionizes DNA molecules (3), or indirectly interacts with water, to form reactive oxygen species (ROS) to induce cell apoptosis (4, 5). Compared to tumor of squamous cell origin, such as the common type of nasopharyngeal carcinoma, breast adenocarcinoma, especially for triple-negative breast cancer (TNBC), has lower sensitivity to X-ray radiation (6, 7). Techniques currently employed to maximize exposure of target volume to radiation include intensity modulated radiation therapy and hypofractionation, but risk of acute and late adverse events still need consideration (8, 9). How to improve the efficacy of RT for triple-negative breast cancer is a question worth pondering.

Radiosensitizers are agents that increase efficacy of RT, and thereby allow for lower doses of radiation and reducing toxicity to organ at risk (10). Notably, clinical trials demonstrated the potential of high atomic number (Z) nanomaterials as enhancer of radiation to target cancer cells (e.g., gold (10–13), bismuth, wolfram (14, 15), platinum, gadolinium (16)) have been exploited. Even though developing radiosensitizers is a promising way to increase the level of efficacy, some difficulties, such as radiation resistance of hypoxic cancer cells, still limit its applications in RT (14, 17). To overcome such obstacles, combining RT with phototherapy (PT) to realize a synergistic therapy can open new potentials (18, 19). Given the limitations of RT alone, new efforts have explored the potential of combining RT with phototherapy (PT), including photothermal therapy (PTT) and photodynamic therapy (PDT), as a synergistic approach to therapy. The photothermal nanomaterial at tumor site creates local hyperthermia under external near infrared (NIR) irradiation (20, 21). The *in vivo* hyperthermia not only contributes to necrosis or apoptosis of cancer cells, but also creates an oxygen-enriched environment, and in effect reduces radiotherapy resistance (22). In combination with PT, lower dose in RT could be sufficient to kill the tumor and simultaneously enhance systemic antitumor immune response, while high dose RT cause damage to immune system (23, 24). Recent evidence has shown that radiation of X-ray and NIR can also be immunomodulatory by altering the microenvironment of the irradiated field (25, 26). Since RT and PT are both local treatments as main role, the anti-tumor immunity effects could compensate disadvantages (24, 27). Although antitumor immunity induced by RT or PT alone is rarely sufficient to activate systemic anti-tumor immune response, whether combination has the potential to extend the applications beyond a local modality is worth investigating.

**Scheme 1:**
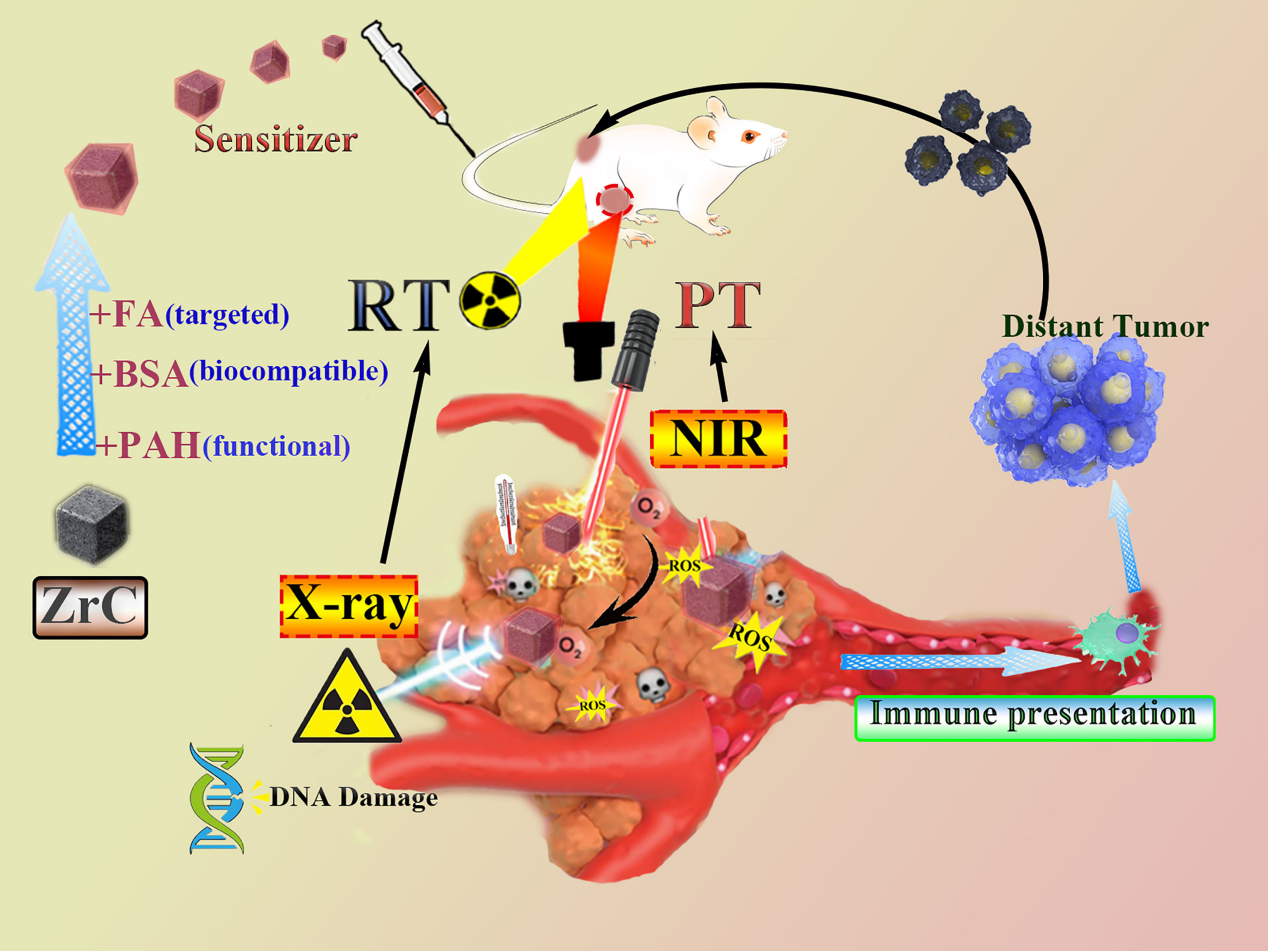
Scheme diagram of radiation therapy combined phototherapy process of this work.

It will be very significant if we can construct an ideal “all in one” nanoparticles to act as the sensitizer for both RT and PT that have the following characteristics: (1) good photo-thermal conversion behavior; (2) can act as radiosensitizers to enhance the deposition of irradiation energy; (3) can be specifically targeted to tumors; and (4) is biodegraded (28). In this work, we present a new multifunctional nano agent to be used in combined PT and RT. Using BSA to modify the nanoparticles in order to improve the biocompatibility, as well as tumor-targeted molecule FA (ZrC-BSA-FA, ZrC NPs). Detailed therapeutic strategy by using ZrC NPs was presented in **Scheme 1**. At the first part, we characteristic ZrC NPs and then tests were performed to see its excellent thermal storage, thermal stability, thermal conductivity and photo-thermal conversion feature. ZrC NPs has been an attractive candidate for PT because of its potential as a dual-modality therapy sensitizer, its efficacy as a radio-sensitizer is investigated here for the first time. At the third part we performed experiments to assess the potential to selectively treat TNBC, including vivo and vitro. To further explain its mechanism, we performed the semiquantitative method to detect tumor apoptosis factors/proliferative factors of tumor. In the last part, we built tumor-bearing mouse model of double tumors to see whether ZrC NPs with PT and RT in mouse could activate immune response *in vitro*. Our results provide proof of concept evidence that the combination of ZrC NPs, PT, and RT is effective for the treatment of TNBC, making it a great potential application for cancer therapy in clinic.

## Materials and Characterizations

### Materials

All chemicals and reagents were used as received without further purification.

ZrC nanoparticles were purchased from Shanghai Chaowei Nano Technology Co. Ltd and stored in a desiccator. Folic acid, Poly (allylamine hydrochloride) (PAH, Mw= 17 500), DCFH-DA and DPBF were purchased from Sigma-Aldrich. Bovine serum albumin (BSA), calcein-AM, propidium iodide (PI), and 3-(4,5-dimethyl-2-thiazolyl)-2,5-diphenyl-2-H-tetrazolium bromide (MTT) were obtained from Summus. All the antibodies were purchased from Abcam. The γ-H2AX antibody was purchased from Abcam. Phospho-Histone γ-H2AX (Ser139) (20E3) Rabbit mAb and Antirabbit IgG (H+L) were provided by Cell Signaling Technology.

BALB/c nude mouse and BALB/c mouse were purchased from Beijing Vital River Laboratory Animal Technology Co., Ltd. Animals are fed sterile water and unrestricted food. They were placed in mice room with standard conditions. All the experimental steps adopted in this experiment conformed to the experimental scheme approved by the Key Laboratory for Biomedical Effects of Nanomaterials and Nanosafety (Chinese Academy Sciences, CAS).

### Surface Modification Process of ZrC Nanoparticles

Prior to the modification, ZrC nanocrystals were heated in NH3atmosphere at 850°C for 2 h for full nitridation. Then, the powder was dispersed in water and underwent ultrasonic dispersion for 20 min. The suspension was centrifuged at 4000 rpm for 5 min to remove bulk particles. The ZrCNPs were prepared *via* electrostatic interaction according to our previously published method with minor modifications. (51), (53) In brief, ZrC(6 mg) and PAH (4 mg) were dissolved in 2 mL NaCl solution (0.5 M) under ultrasonic dispersion for 20 min with a mass ratio of 1: 4. Then, the sample was collected by centrifugation (12 000 rpm, 5 min) and rinsed three times with NaCl solution (0.1 M) to obtain ZrC@PAH.

After that, ZrC@PAH and BSA with a mass ratio of 1: 3 were dissolved in deionized water and continuously stirred for 4 h in an ice bath. Finally, the suspension was centrifuged, washed with distilled water and freeze dried, obtaining ZrC@PAH/BSA.

After ZrC@PAH/BSA were prepared, they were mixed with 0.5% (wt/vol) of FA–gelatin solution and stirring at room temperature for 24 hours. The FA-functionalized ZrC@PAH/BSA/FA were collected by centrifugation, washed with Milli-Q water, and redispersed in Milli-Q water for further use.

### Characterizations

The nanostructural features of sample were observed on a JEOL 2100 electron microscope at 200 kV (JEOL Ltd.), including transmission electron microscopy (TEM). The optical behavior of the samples was measured on a spectrophotometer (U-4100, Hitachi). TEM observation was performed using a FEI Tecnai G2 F30 system. The nanoparticles characteristics were determined by XRD (Shimadzu XD-D1). XPS spectra (PerkinElmer PHI 5600) were used to analyze the composition and chemical valence of the samples. The absorption spectra were performed by Cary Series UV-Vis-NIR Spectrophotometer. The hydrodynamic diameter was measured by a Zeta-Sizer (Malvern Nano series). FTIR was acquired by the Excalibur 3100 (Varian). Temperature monitoring was realized with an infrared camera (FLIR System i7).

### Photothermal Test

To examine the photothermal performance of heterogeneous ZrCNPs, different concentrations of CSA solutions were prepared and heated with an 808 nm laser at different power. An infrared thermal imaging camera (FLIR, A65) was applied to record the change of temperature. The photothermal stability of ZrCNPs was also examined by heating and cooling for 5 cycles. For each cycle, 808 nm laser was turned on to heat the ZrCNPs solution for 10 minutes and then switched off to allow cooling to room temperature.


 (1) $$\eta =\frac{\text{hS}({\text{T}}_{\max}-{\text{T}}_{\text{surr}})-{\text{Q}}_{\text{d}}}{\text{I}(1-10^{-\text{A}}808)}$$

In the equation 1, S is the surface area of the container, h is the heat transfer coefficient, Tmax and TSurr are the equilibrium temperature and ambient temperature of the surroundings, respectively. Qdis is heat dissipated from light absorbed by the quartz sample cell itself, I is the incident laser power (1.0 W cm-2) and A808 means the optical absorbance of ZrC NPs at 808 nm. Only the hS remains unknown. We introduced a dimensionless driving force temperature to obtain the value of hS.


 (1.1) $$\theta =\frac{\text{T}-{\text{T}}_{\text{surr}}}{{\text{T}}_{\max}-{\text{T}}_{\text{surr}}}$$

(1.2) $$\text{t}=-\tau_{\text{s}}\ln(\theta )$$

(1.3) $$hS=\frac{\Sigma m_{i}C_{p,I}}{\tau_{s}}$$

Equation 1.1 shows the cooling stage of the aqueous dispersion, the cooling time t and abide by the equation 1.1, time constant (τs) for heat transfer from the system could be determined.

Where m, Cp,I are the mass, heat capacity of water, respectively. According to Equation S1.1, Equation S1.2 and Equation 1.3, the value hS is obtained. The photothermal conversion efficiency (η) is calculated to be 20.51% according to Equation 1.

### Cell Lines and Cell Culture

4T1 cells were routinely cultured in DMEM (Corning) medium containing 10% (v/v) FBS (Gibco), and 1% antibiotics (penicillin–streptomycin) (Corning) in a humidified atmosphere at 37°C including 5% CO2.

### Cytotoxicity Assay

We performed MTT method to illustrate this issue. 4T1 cells and HUVE cells were seeded into 96-well plates (1 × 104cells per well) and co-incubated with the ZrCNPs dispersion of different concentrations for 24 hours. Then, 20 μL (5 mg/mL) MTT solution was introduced into each well and incubated for another 4 hours. After that, the medium was replaced with dimethyl sulfoxide (DMSO, 150 μL per well) for 30 min. A microplate reader (SynergyTMHT, BioTek Instruments Inc., USA) was used to test the optical absorbance at 490 nm.

### *In Vitro* Phototherapeutic Effect

To explore the optimum sensitizer dose of PTT and RT, the *in vitro* cell survival rate was evaluated on 4T1 cells using a standard MTT assay. The experiment controlled for a single variable at a time and we draw a conclusion:

For RT, cells were treated by linear accelerator of image guided radiotherapy, X-ray radiation set was 6MV, 4Gy and 300 Does Rate. The height of the liquid level from the underlying cells is 1 cm. For PTT, cells were treated by 808 nm laser at power density (2.0 W cm-2). They were divided into six groups, including control, NIR, ZrC+NIR, X-ray, ZrC+X-ray. ZrC+NIR+X-ray All the experiments were performed for three times. 4T1 cells were seeded into a 35 mm culture dish (3 × 105 cells per dish) and incubated at 37°C until being nearly 90% coverage. The culture medium containing ZrC NPs (250 µg mL-1 was added into the culture dish and incubated for 6 h). After washing with PBS for three times, the fresh culture medium was added into culture dish and the cells were treated. Afterward, the cells were stained using cell double­staining method (calcein­AM and PI) for 20 min to distinguish the living and dead cells, and then observed immediately using fluorescence microscope (Olympus BX53)

### Cell Experiments (γ-H2AX Antibody Recognition Immunofluorescence)

The cells and anti-phospho-histone γ-H2AX mouse monoclonal antibody (dilution 1:1000) were incubated together overnight at 4°C. Next day, the sheep anti-mouse secondary antibody (dilution 1:1000) was incubated with the cells at room temperature for 1 h. DAPI was used to stain cells by labeling the nuclei at room temperature for 5 min. Eventually, the Leica SP5 confocal microscopy was used to observe the cells.

### Detection of ROS

Intracellular ROS generation was detected using DPBF and H2DCFH-DA probe, which could be oxidized to produce fluorescent compound of DCF in the presence of ROS. 4T1 cells were seeded into a 35 mm culture dish and incubated overnight. Afterward, the medium was discarded and cells were incubated with fresh medium containing ZrC NPs (0.5mg mL-1) for 4 h. The cells were then washed with PBS. Untreated cells were used as negative control. After that, the cells were stained with H2DCFH-DA (50 µL, 10 × 10-3m) for another 1 h. Thereafter, the cells were washed with PBS and treated in 6 groups as above. The DCF fluorescence images were acquired by fluorescence microscope (Olympus BX53).

### Calculation of the Sensitization Enhancement Ratio

Cell survival fraction of each group was calculated by the ratio of the seeded cells following treatments to form colonies versus the untreated cells as described above. Cell survival fraction and sensitization enhancement ratio (SER) were determined by a classical multitarget single-hit model.and the SER was expressed with the following formulas:


$$\text{SER}=\frac{{\text{D}}_{\text{q}}(\text{control group})}{D_{q}(\text{sensitizer group})}$$

$$D_{q}=\ln(n)\times D_{0}$$

$$\text{S}=1-{\left(1-{\text{e}}^{-\frac{\text{D}}{{\text{D}}_{0}}}\right)}^{\text{n}}$$

where S is the survival fraction, D is the radiation dose, D_0_ is the mean lethal dose, and n is the extrapolation number. Where D_qis_ the quasi-threshold dose.

### *In Vivo* Antitumor Effect

1×10^6^ 4T1 cells were suspended in 100 µL PBS mixed with matrix glue 1:1 (V:V), and were inoculated at the left hind legs of female BALB/c nude mice. The tumor-bearing mice were then divided into six groups (five mice each group) when the tumor was about 200 mm^3^in size: (a) control, (b) NIR alone, (c) ZrC NPs+NIR, (d) X-ray alone, (e) ZrC NPs +X-ray and (f). ZrC NPs +NIR+X-ray. The 4T1 tumor-bearing mice from groups (c), (e), and (f) were administrated nanomaterials intravenously, and the other 3 groups were injected with PBS instead as control. Immobilization scan is operated with Philips CT sim for RT. And RT for mice was performed by Varian IX medical electron linear accelerator immediately after PT. Then, mouse weight and tumor growth were measured after for 14 days. The length and width of tumor were measured with a vernier caliper daily. When the whole experiment finished, all of the mice were sacrificed according to the protocols of Harbin Medical University Cancer Hospital. The tumor and major organs were removed and weighed to calculate the therapy efficacy among six groups.

To study whether the immunity could be triggered by the treatments and how strong it could be on suppressing distant tumors, we designed two experiments to evaluate the therapeutic effect as shown in **Figures 6A, E**. Two groups of the 4T1 tumor-bearing mice were divided randomly: 1) control 2) ZrC treated (ZrC+N+X). In experiment A, we built tumor-bearing mouse model with bilateral tumors at -13th day (n = 5 for each group), and treated primary one (left) at day 0, leaving the other side distant tumor to be untreated. Thereafter, the tumor volume changes of distant tumor were recorded. The tumor growth rate showed no significant difference between the two groups. In experiment B, we implanted the tumor seeds on the other side of mice post treatment and measured their growth rate. As be seen in **Figures 6B, F**, although the post treatment-implanted tumor grew not as fast as before, distant tumors on treated mice showed more limited tumor growth rate. The logic explanation of the study is the antitumor immune responses of host.

### Immunohistochemical Examination

Immunohistochemistry (IHC) was performed according to the instructions from manufacturer to detect the antibodies, apoptotic factor, multiplication factor, and hypoxia factor in tumor. The anatomical operation of the organs according to standard histological techniques and all the tumor tissues were frozen and then made into 4 µm thick serial sections. These paraffin sections were used for IHC staining using standard method. The pathologist was blinded to the identities and analyses of the pathology slides.

### Western Blot Analysis

Total protein was purified and extracted from tissues with RIPA buffer supplemented with proteinase/phosphatase inhibitors (Thermo, Cambridge, MA). Same amounts of total protein were resolved by 10% SDS-PAGE, followed by transfer onto nitrocellulose membranes (Millipore, Bedford, MA). Anti-Bax (1:500), Bcl-2 (1:500), caspase (1:500), Ki67(1:500), HIF-1α(1:500(Abcam, USA) primary antibodies were used for the detection

### Flow Cytometric Analysis

Tumors and surrounding tissues were obtained after combined therapy (be specific here). Phenotyping of cells was stained with the following antibodies: FITC-CD3, PE-CD4, PC7-CD8 (BD Phar Mingen, San Diego, CA). Flow cytometric analysis was performed with FACS Calibur (BD Biosciences, Franklin Lakes, NJ)

### Histology Analysis

The mice were sacrificed to collect the tumors and major organs (heart, liver, spleen, lung, and kidney), which were fixed in 4% paraformaldehyde and embedded with paraffin. The slices of the major organs and tumors of the mice were stained with H&E for histological analysis. Finally, optical microscope images were acquired by a fluorescent microscope (Olympus BX53).

### Hematological Analysis

Blood (100 µL) specimen from the mice were collected and analyzed at 14th day using automatic blood analyzer (HF­3800).

Statistic calculated using “Statistical Program for Social Sciences” software (SPSS, version 19.0) and graphpad Prism 8. The final survival fraction curve of each group was generated *via* a nonlinear fitting using Origin 8.0.

## Result

### Preparation and Characterization of ZrC NPs

In this work, we selected ZrC nanoparticles as the sensitizer to be used in conjunction with RT and PT. To enhance the biocompatibility of ZrC, we alternately coated polyallylamine hydrochloride (PAH, positively charged) and bovine serum albumin (BSA, negatively charged) on the surface of ZrC *via* electrostatic interactions. In addition, we used folic acid (FA) for surface modification of the nanoparticles to increase tumor targeting by enhancing the utilization of sensitizer *in vivo*. The initial zeta potential of unmodified ZrC nanoparticles is 23.8 mV, and after modification with PAH-BSA-FA, the potential changed to 8.61 mV, -12.98 mV, and 8.47 mV. The change in zeta potential indicates the successful surface modification. This is also supported by the increase of their hydrodynamic size from 95.9 nm to 121.2 nm (**Figure S1**). To determine the dimension of ZrC NPs in the aqueous solution, the hydrodynamic diameter of ZrC NPs was tested *via* dynamic light scattering (DLS) measurement and determined to be 89.6 ± 1.6 nm (**Figure S2**), which is a suitable size for further *in vivo* administration. The PDI value corresponding to the DLS result is 0.187. As shown in **Figure S3**, A is the ZrC powder dissolved in PBS and B is the solution of ZrC NPs. Both solutions were allowed to stand for six hours, and A showed observable precipitation while no obvious change in B, indicating their better stability in physiological solutions.

**Figure 1:**
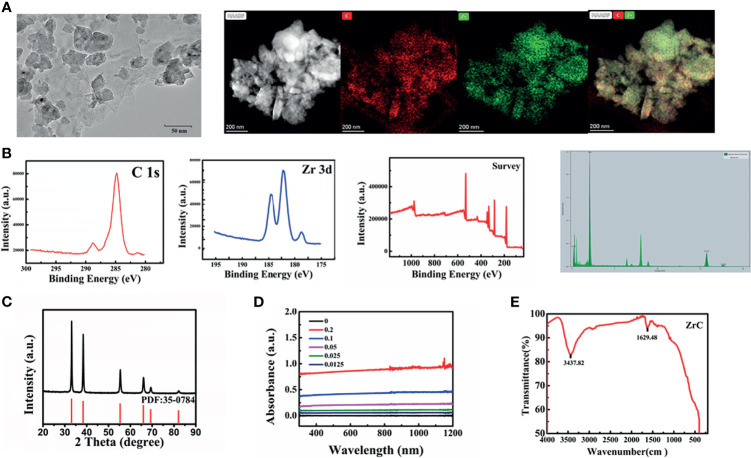
**(A)** The TEM image and elements mappings image, **(B)** XPS, **(C)** XRD PDF:35-0784, **(D)** UV spectra: optical absorbance of ZrC dispersions with varied concentration, **(E)** the FTIR spectrums.

The TEM image of ZrC is presented in **Figures 1A** and **S4**, showing that the ZrC nanoparticles are about 35 nm in size. The high-resolution TEM (HRTEM), elemental mapping and energy dispersive spectrometer (EDS) were used to further characterize the material. The results demonstrate that the nanoparticles consist of Zr and C elements, as proven by an overlay of green (Zr) and red (C) colors in each particle. Next, the chemical valence of the as-prepared ZrC NPs was determined *via* XPS analysis **Figure 1B**. All reflective peaks are well matched with standard cubic ZrC. After deconvolution, the XPS core-level of Zr 3d orbit results disclose the presence of Zr2+, Zr3+ and Zr 4+ ions in the sample. Classically, the XRD **Figure 1C** pattern reveals that all the diffractive peaks are attributable to standard cubic ZrC (PDF#35-0784). Additionally, **Figure 1D** shows the powder UV-Vis-NIR spectra of ZrC. ZrC powder exhibits strong and broad absorption in the whole NIR window. Next, FTIR **Figure 1E** spectrum was applied to detect the chemical compositions of ZrC NPs.

### Photothermal Properties of ZrC

**Figure 2:**
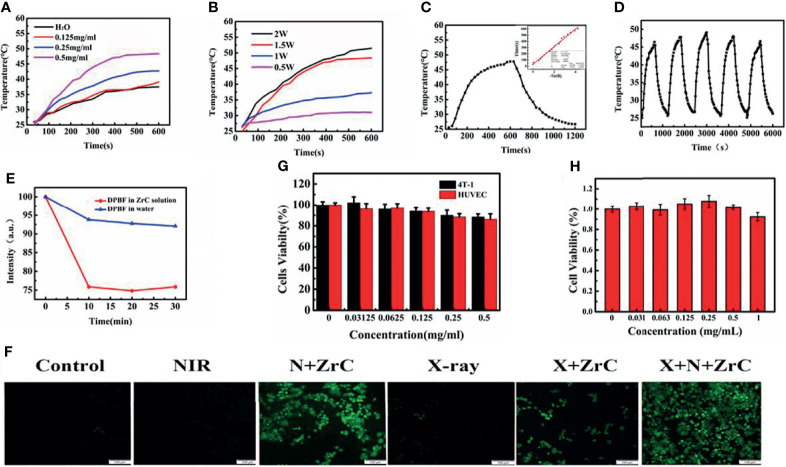
**(A)** Photothermal temperature curves of ZrC NPs aqueous dispersions at different concentrations. **(B)** Photothermal temperature curves of ZrC NPs aqueous dispersions at different power of NIR laser. **(C)** Photothermal test of ZrC dispersion of 0.5 mg·mL-1. (NIR laser: 808 nm, 1.5 W·cm-2; inset shows plot of time versus – ln (Ɵ); The ZrC dispersion was irradiated with NIR for 10 min and left to natural cooling for another 10 min), **(D)**Temperature change of ZrC NPs solution at concentration of 0.5 mg·mL-1over five laser on/off cycles. (808 nm laser irradiation, with a power density of 1.5 W cm-2. **(E)** DPBF degradation behavior for ROS detection (NIR laser: 808 nm, 1.5 W·cm-2); **(F)** Confocal fluorescence images of reactive oxygen species (ROS) in 6 groups. **(G)** *In vitro* cytotoxicity of different concentrations of ZrC NPs to 4T1 cells and HUVE cells, **(H)** The biocompatibility of ZrC NPs on proliferation ability of 4T1 cells at different concentration (0, 0.03215, 0.0625, 0.125, 0.5 and 1 mg/ml) after incubation for 48 h. Error bars represent mean ± SD. **(F)** The cytotoxicity of ZrC NPs on 4T1 and HUVE cells at different concentration (0, 0.03215, 0.0625, 0.125, 0.5 and 1 mg/ml) after incubation for 24 h.

Photothermal therapy requires photo absorption in the NIR region and the resultant photothermal properties of photoactive materials is particularly important. We first inspected the optical absorption properties of the ZrC aqueous dispersion by correlating the change in temperature with the concentration of ZrC over an irradiation time. Different concentrations of ZrC NPs (0.125, 0.25, 0.5 µg/mL) were heated by an 808 nm NIR laser with various power (0.5,1,1.5,2 W cm-2) for 10 min, and the transformation in solution temperature was recorded by an infrared thermal imaging camera. As shown in **Figure 2A**, pure water and ZrC dispersion of 0.125 mg/mL showed no significant temperature elevation (about 10°C) after 10 min of NIR irradiation. In contrast, the temperature of the ZrC dispersion of 0.25 and 0.5mg/mL increased significantly. The curve in **Figure 2B** reveals the relationship between temperature increment on the nanoparticle concentration and power, the maximal temperature reached 42.3 and 47.6°C for 0.25 and 0.5 mg/mL ZrC dispersion, respectively. The photothermal conversion efficiency was calculated to be 40.51% (**Figure 2C**, the calculation details are presented in method and material). The optical density at 808 nm had a good linear relationship with concentration of ZrC NPs (the inset of **Figure 2C**). In addition, the temperature change of ZrC NPs solution upon NIR on/off irradiation did not change much for five rounds. Shown in **Figure 2D**, our results revealed photostability of ZrC NPs. Moreover, ZrC also has a very high absorption in the NIR region, which improves the infrared absorption value of ZrC NPs to some extent. Such photothermal production and photostability indicated that ZrC can act as an efficient photothermal conversion agents(PTA).

Inspired by the previous study of X-PDT and PDT, we investigated ROS production from ZrC NPs under NIR and X-ray irradiation by using sodium terephthalic as a probe. The 1,3-diphenylisobenzofuran (DPBF) and 2′7-dichlorofluores-cin diacetate (DCFH-DA) probes were employed to detect the extracellular and intracellular ROS production, respectively. The DPBF probe for detecting extracellular ROS production lies in its decomposition by ROS. As result, a decrement of DPBF characteristic absorption could be observed accordingly. As displayed in **Figure 2E**, the absorbance of the DPBF solution with ZrC decreased meaningfully as irradiation time and X-ray dose increased, suggesting that significant level of ROS was induced by ZrC under NIR and X-ray. In contrast, the pure water led to very limited ROS generation with the same laser irradiation.

We examined intracellular ROS production in 4T1 cells with the use of DCFH-DA probe, which would convert to green fluorescent molecules after being oxidized by ROS, to detect the presence of ROS. As shown in **Figure 2F** there was no observable green fluorescence from the negative control, while a very weak signal was observed from the NIR group and X-ray group. The fluorescence intensity of sample irradiated by both X-ray and NIR, contained ZrC NPs, increase 75% than that of the average of other 5 groups, indicating the ROS producing ability of ZrC NPs. This result is in agreement with the above-mentioned photocurrent experiment. Therefore, the as-prepared ZrC NPs have potential as both radiosensitizers and photosensitizers for tumor treatment.

### *In Vitro* Cell Cytotoxicity and Phototherapeutic Treatment Combined Radiotherapy

To determine the optimal nanoparticle concentration for subsequent experiments, we investigated cytotoxicity towards mouse breast cancer 4T1 tumor cells and normal human umbilical vein endothelial cells (HUVECs) using standard MTT assay. was investigated. Cell viabilities are strongly dependent on the concentration of ZrC NPs. There were no significant differences within or between groups and more than 80% of the 4T1 cells, and HUVECs survived when they were cultured with 0.5 mg/mL ZrC NPs solution (**Figure 2G**). **Figure 2H** demonstrates the effects of different concentration of solution on proliferation of 4T1 cells, and then MTT was performed in 48 hours. As shown in the histogram, there was no significant change between the groups, signifying the minimal effect of ZrC NPs on the cells’ proliferation ability.

**Figure 3:**
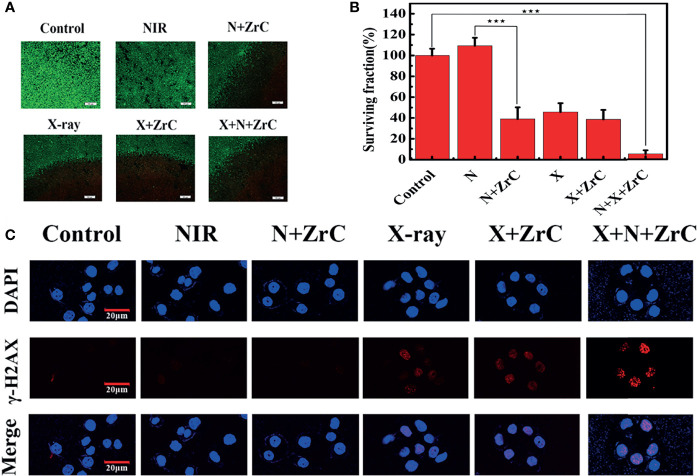
**(A, B)** live/dead images of 4T1 cells and survival rates analysis after different type of treatment with six groups respectively: Control, NIR, N+ZrC (PT+ZrC NPs), X-ray, X+ZrC (RT+ZrC NPs), X+N+ZrC (RT+PT+ZrC NPs) in concentrations of 0.5 mg mg·mL-1and irradiated with an 808 nm laser 4min with 1.5 W cm-2, X-ray with 6MV,4Gy. Error bars represent mean ± SD. P values based on Student’s t-test: ^∗∗∗^p < 0.001. **(C)** Qualitative representation of DNA fragmentation with different treatment (scale bar: 20 μm).

The cytotoxicity results suggest that the concentration of ZrC NPs applied to tumor cells should be no higher than 0.5 mg/ml. To detect the effective energy level of X-ray for cell experiments, 4MV, 6MV and 12MV were selected with various radiation dose (2,4,6,8Gy). The results show 6MV group had the statistically significant difference (about 43.47%) as demonstrated in **Figure S5**. From S6, based on the obvious difference of cell surviving fraction between sensitization of ZrC NPs (25.3% in 4Gy), 4Gy of RT with 6MV was used for the experiment. PT, 4min with 1.5 W cm-2 was selected, the differences of surviving fraction was 28.6%. We discovered that the final survival rate in these conditions were about 40% after sensitization. If the single treatment kills most of tumor cells, it will be meaningless to combine additional treatment. Basing on these explorations, we started to compare the killing effect of different therapeutic methods on tumor cells. As shown in **Figure 3A**, a Live/Dead kit was used to test the activity of 4T1 cells in 6 groups, in which the living cells were stained green and dead cells were stained red. Notably in **Figure 3B**, with the data analysis of MTT, ZrC NPs played an effective role in sensitization of PT (surviving rate from 109.32% to 39.01%) while RT(from 45.71% to 38.66%), and the combination therapy of PT/RT with ZrC NPs contributed to the best cancer cell killing efficiency, where the cell survival dropped to 5.16%, demonstrating the powerful synergistic PT and RT effects *in vitro*. There is a result does not seem to meet expectations that the cell surviving fraction in NIR group was higher than the control group. This is because all the groups were treated under the same conditions, and the room temperature was relatively low during radiotherapy, so cell survival rates were higher with mild PTT. The sensitizer enhancement ration (SER) in this experiment was used to evaluate the radio sensitization efficiency. The SER value of ZrC+N+X group was about 1.8 compared with X-ray group, further confirming that PT *via* ZrC NPs can promote RT function on the cancer cells. However, ZrC+X group’s SER is about 1.2. Analyze from these data, we found that higher temperature around tumor induced by ZrC NPs under NIR had obvious sensitization effect on RT while ZrC NPs alone showed weak sensitization effect.

The radiation enhancement was confirmed to be attributed to the process of DNA breaking. **Figure 3C** showed there were no significant γ-H2AX fluorescent signals in control and NIR treated groups. Only limited γ-H2AX spots in fluorescent were detected in N(ZrC, X-ray, and X+ZrC groups. Comparing to other groups, a very high amount of γ-H2AX foci was detected in N+X+ZrC group, indicating significant more DNA breaking due to enhanced X-ray generation by synergistic treatment. There results suggest that ZrC NPs mediated synergistic treatment provides an approach to kill cancer cells with high efficiency. These results were consistent and demonstrated the possibility of using sensitization of PT combined RT in tumors and leading to DNA breakdown in tumor cells.

### *In Vivo* Investigation Anti-Cancer Efficacy

**Figure 4:**
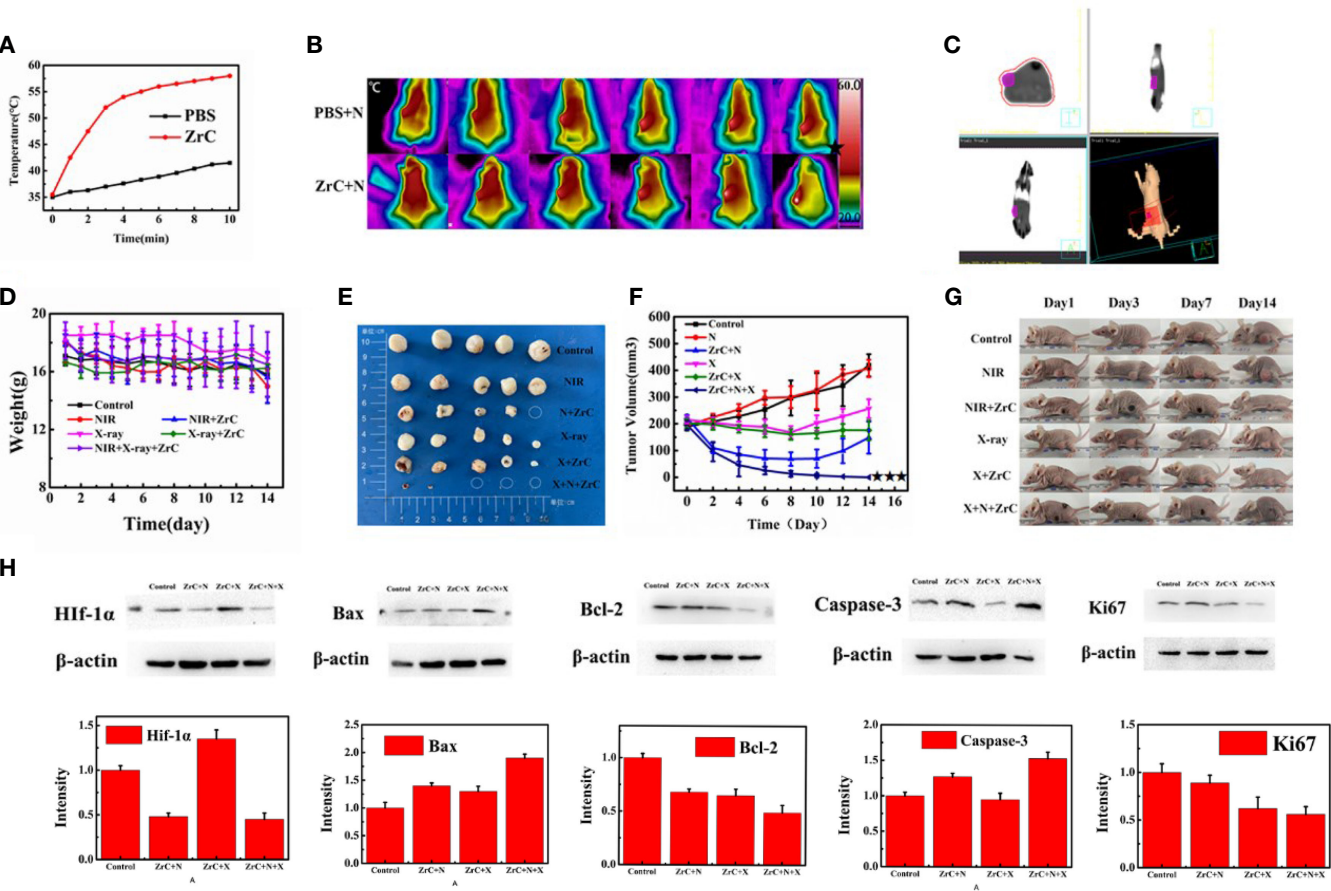
**(A)**The temperature curves of the tumor site after they were intravenously injected with ZrC NPs (dose 0.5 mg mL-1, 200 µL) and PBS, respectively, and then exposed to an 808 nm NIR laser (1.5 W cm-2, 10 min); **(B)**Infrared thermal images of tumor-bearing mice while PT. **(C)** RT plan for mice. **(D)** The variations in weight of mice in six groups. Error bars represent mean ± SD. **(E)** Tumors were cut out at 14 days after treatment from five mice in each group. **(F)** Tumor volume in six groups were measured after treatment. Error bars represent mean ± SD. **(G)** Representative photos of tumor-bearing mice after 14 d treatment. **(H)** In control, NIR(PT), ZrC+N (PT+ZrC NPs), X-ray (RT), ZrC+X (RT+ZrC) and ZrC +N+X (RT+PT+ZrC NPs) groups, respectively. Protein expression measured by Western Blot of HIF-1a/Bax/Bcl-2/Caspase-3/Ki67 and Intensity expression level of these genes.

We further investigated the *in vivo* photo-therapeutic efficacy of ZrC NPs *via* a subcutaneous 4T1 tumor-bearing mice model. When the tumor volume reached≈200 mm3, 4T1 tumor-bearing mice were randomly divided into six groups (n = 5 for each group) for various treatments: group 1) Control, 2) NIR alone (PT), 3) ZrC +NIR (PT+ZrC NPs), 4) X-ray alone (RT), 5) ZrC+X-ray (RT+ZrC),6) ZrC+NIR+X-ray (RT+PT+ZrC NPs). The Balb/c nude mice were intravenously (i.v.) injected with 100 µL of PBS (group 1) or ZrC NPs (group 3, 5, 6) and then exposed to NIR irradiation (808 nm, 1.5 W cm-2 10min) or X-ray (6MV, 6Gy) at 2 h post injection for group2,3,4,5 and 6. First, the surface temperature profiles of tumor region were recorded using an infrared thermal camera during PT at specific points in time. As illustrated in **Figures 4A–B**, the temperature of tumor site with ZrC NPs administration rapidly increased to about 57.3°C within 10 min under NIR laser irradiation. However, the temperature of tumor site with only NIR laser irradiation exhibited limited temperature (from 35.5°C to 41.5°C) elevation under the same conditions. As temperature increased very limited after 6 min and this is the meaningful point to be sensitized, so PT’s time is settled to be 6 min in mice treatment. Shown in **Figure 4C**, it is the RT plan for mice. 14 days post-treatment for the body weight and tumor volume. No significant difference was observed in body weight among six groups. However, groups got X-ray irradiation lose a little more weight, most likely due to intestinal mucosa injury after RT when the black mice feces were noticed for about 3 days **Figure 4D**. To evaluate tumor response, the tumor volume was measured in each group within 14 d. From the comparison of group 2 and 3, group 4 and 5, it shows stronger antitumor effect in the later groups which were injected with ZrC NPs, indicating that ZrC NPs played a sensitizer role in both PT and RT, respectively. The relative tumor volume of the mice treated with RT or PT alone was slightly smaller than that in the control group, which illustrated that RT and PT could mildly inhibit the growth of the tumors. However, the mean tumor volume of group 6 was the lowest in all six groups (**Figures 4E–G**) which indicated that the ZrC NPs as sensitization materials enhanced PT and RT efficacy and led to substantial better tumor control. Remarkably, although the tumor volume recorded after treatment showed same trend on ZrC+N group and ZrC+N+X group in the first few days after treatment, remnant tumor tissues were noticed beneath black scab in ZrC+N group as a common phenomenon and showed high local recurrence rate after a week, while in ZrC+N+X group: the interior of the tumor is dominated by residual pus and necrotic tissue.

**Figure 5:**
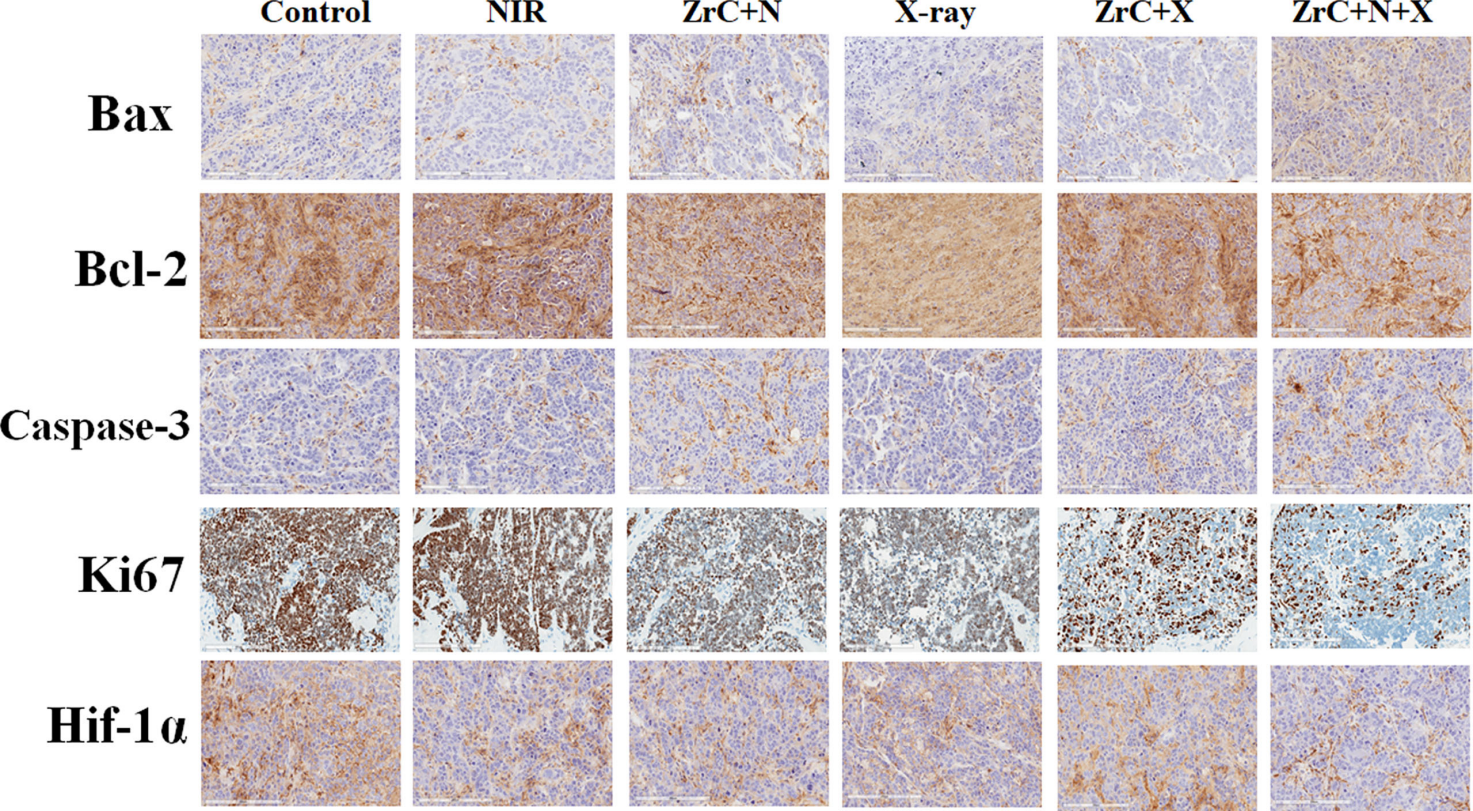
Immunohistochemical analysis of related genes of tumor tissues from six groups of 4T1 tumor-bearing mice after treatment. Furthermore, H&E staining on the major organs, including heart, kidney, lung, liver and spleen, disclosed no notable damages (i.e., inflammation or necrosis) of all mice (S12). Because of the tumor targeting effect of ZrC NPs, the materials concentrate around the tumor. They result more energy deposition from NIR and X-ray interactions and protect normal tissues while achieving the strongest tumor killing. These results showed a very promising potential which can significantly improve the limitation of single therapy regimen by altering the tumor microenvironment, achieving a safe and efficient elimination of tumor cells.

After PT/RT treatment, the mice show the black scars at their original sites of tumor. To give a better visualization of the killing of tumor, the mice were sacrificed 14 d later after therapy and histology analysis of tumor tissues was performed *via* a typical hematoxylin and eosin (H&E) method. SI.8shows massive tumor cells necrosis in group ZrC +N and ZrC +N+X, but not in other four control groups. To provide a deep insight into the mechanism of tumor-growth inhibition, the expression level of bax, bcl-2, caspase-3 which were associated with apoptosis, and proliferation related gene Ki67 were tested by immunohistochemical analysis of tumor tissues and western blot, respectively. As illustrated in **Figures 4H**, **5**, ZrC+N+X group had higher expression of bax and caspeas-3 and lower expression of bal-2, both associate with cell apoptosis. To evaluate the ability of tumor proliferation, the expression of Ki67, representing the proliferation ability of cells, was examined. We found that the expression level of Ki67 in the combined group was lower. Also, the ability of proliferation in tumor was not inhibited by any single treatment compared to sensitization therapy groups. From the expression of western blot, a conclusion could be drawn that in the groups with X-ray, the proliferation factors showed lower expression while apoptotic factors showed higher in PTT and combined therapy groups, owing to X-ray dose damage to the DNA double-stranded structure and local heat leads to a disruption on cell membrane permeability fist. To further verify the previous hypothesis that the combination of PT and RT could sensitize RT by improving tumor hypoxic microenvironment. Hypoxia-inducible factor 1a (Hif-1α) was tested by western blot and Immunohistochemical analysis from tumor and surrounding tissue. HIF-1α often expressed in tumors and its transcriptional activity is accurately regulated by the concentration of oxygen, meaning that the stronger Hif-1α expressed the more severe of hypoxia is. In, it showed that the protein expression of Hif-1α in group ZrC+N was the lowest, and slightly higher in group ZrC+N+X while high in control/X-ray alone/ZrC NPs+X groups. The expression in group ZrC+N+X was the highest, meaning the RT consumes oxygen in tumor microenvironment produced by PT. The same expression trend of Hif-1α showed up in immunohistochemical analysis. As the tumor growth inhibition rates were positive correlated to absorption doses and the improvement of hypoxic environment can promote the absorption of radiotherapy (22, 29). To summarize the above experimental data *in vivo*, a conclusion can be drawn that the combination of PT and RT with ZrC NPs alleviate hypoxia induced resistance of RT and sensitizing tumor tissues to absorb more doses of PT and RT, improving cell apoptosis and tumor suppression rate.

### *In Vivo* Evaluation of Immunoassay

**Figure 6:**
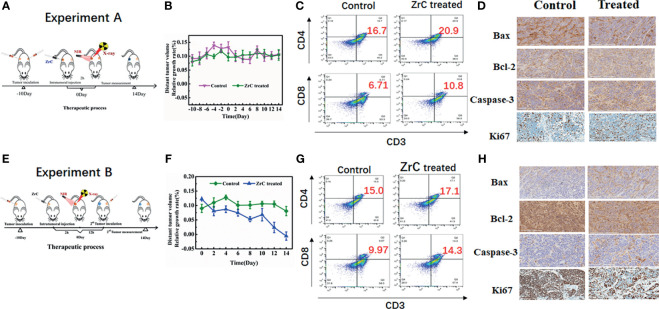
**(A, E)** Schematic illustration of A/B experiment design and measure the distant tumor volume. **(B, F)** Relative growth rate of distant tumor volume in two groups. Error bars represent mean ± SD. **(C, G)** Representative flow cytometry plots showing different types of T cells in tumor tissue from different groups of mice. **(D, H)** Immunohistochemical analysis of tumor tissues.

Recent studies reveal that the free radicals generated by NPs, as well as necrotic tissue debris after PT, are capable of increasing tumor immunogenicity, and thus making these materials possible for cancer immunotherapy (30, 31). To study the benefits of ZrC NPs mediated PT and RT, we explored the immunity effect of the material in post treatment mice by: 1) measuring the growing rate of distant tumor on the other side of the body 2) investigating populations of T cells, analyzed with flow cytometry. 3) immunohistochemical analysis of apoptosis, and proliferation related gene of the distant tumors. The results of the distant tumor inhibition experiment showed that the immune response induced by combined therapy an inhibition effect on new metastatic tumor growth rate after treatment, but had no certain effect on existing distant tumors (32) (**Figures 6A, B, E, F**, **S9**). Besides, the first implanted tumor could stimulate some immunity *in vivo* to some extent.

In addition, we verified above assumption of immune responses further by analysis of flow cytometry. In (**Figures 6C, G**), the immune cells (tumor-infiltrating cytotoxic T lymphocytes CD8 (CD3^+^CD4^−^CD8^+^) and helper T lymphocytes CD4 (CD3^+^CD4^+^CD8^−^) were analyzed in untreated distant tumors from different groups. In experiment A, CD4 and CD8 raised slightly in ZrC treated group. Relatively, the proportion of CD8 raised more in experiment B while CD4 raised less.

Immunohistochemical analysis of bax, bcl-2, caspase-3 and Ki 67 were examined from distant tumor tissues. Shown in **Figures 6D, H**. there was no obvious difference between two groups in experiment A. However, in experiment B, similar to caspase-3, bax showed a higher expression in the treated group while bcl-2 showed a lower expression, indicating the enhanced immune system which promoted cancer cell apoptosis. Expression of Ki6 showed the following difference: higher in control group and lower in combined therapy group, confirming that combined treatment impacted the ability of distant tumor proliferation.

### *In Vivo* Safety Evaluation of ZrC NPs

Lastly, we evaluated the safety of nanomaterials for biomedical application.

No significant abnormal behavior, such as twitching, drowsiness, hobbling, or weight loss were observed in the mice throughout the entire experiment period. No obvious difference was observed in routine blood data after treatment by nanomaterials. To further confirm the treatment safety, major organs of mice from each group were harvested and cut into slices for histochemical analysis through staining with H&E. As illustrated in S12, histological study of major organs (heart, liver, spleen, lung, kidney) showed no obvious tissue damage or side-effect in all treatment groups. As for in the blood biochemistry index, besides routine inspection, we measured the hepatic and renal function markers including ALT, AST, BUN and CREA. We separated the mice into 2 groups depending on the application of ZrC NPs or not. One group included: 1) control, 2) NIR alone, 4) X-ray alone, the other group included:3) ZrC +N,5) ZrC+X,6) ZrC+N+X. The white blood cell count increased in group with ZrC NPs and that was expected as ZrC NPs are foreign bodies in the blood that stimulate white blood cell growth. Besides, serum biochemical examination showed slightly increased in groups with ZrC NPs, corresponding no noticeable renal and hepatic dysfunction induced by the application of ZrC NPs, the results showed the slightly increase in S13-14 All results showed that ZrC NPs are safe *in vivo*.

## Discussion

In TNBC, RT alone has limited curative effect because of the insensitivity of cancer cells to X-ray radiation. To enhance the effect of treatment, this work is the first to present the synthesis and application of brand-new ZrC NPs as sensitization substance for PT/RT combination therapy. In this way, to kill the tumor, less does of X-ray and less time of NIR could be enough. Due to the strong photo absorption capability in the NIR region, the synthesized ZrC NPs can generate thermal energy and ROS upon NIR irradiation, simultaneously enhancing RT therapeutic effects. The combined therapy of PT and RT has shown great potential to produce excellent antitumor outcome. Immunohistochemistry, histopathological analysis and western blot were utilized to demonstrate ablation mechanism of tumor. Biocompatibility, ZrC NPs mediated therapy showed no obvious hematotoxicity and systemic toxicity. In addition, combination regimen makes it possible to deliver lower dose PT and RT, relatively, which could avoid the side effects of a single, intense treatment on patients, such as the damage for immune system from high dose RT. Without this drawback, the combination triggered the immune effect and induce tumor immunity for the treatment of metastatic tumors. The investigation attempts to provide some insight into the treatment of TNBC, not only in the sensitization of RT, but also the multiple combinations of treatment.

## Data Availability Statement

The original contributions presented in the study are included in the article/**Supplementary Material**. Further inquiries can be directed to the corresponding authors.

## Ethics Statement

This study was approved by the institutional research ethics committee of Harbin Medical University. All animal experiments were performed in strict accordance with the ARRIVE guidelines and were approved by the institutional Animal Care and Use Committee of Harbin Medical University.

## Author Contributions

SJ and ZL worked at the sequence alignment, animal experimental, and drafted the manuscript. YT and YG carried out the immunoassays and nanomaterial characterization. SJ, MZ, and SP participated in cell experimental and performed the statistical analysis. HH carried out the plan of radiation therapy and dose calculation. HH and WC joined the study’s design and performed the statistical analysis. AT participated in the article’s text polish and data integration. SJ and ZL contributed equally to this study. All authors contributed to the article and approved the submitted version.

## Funding

This work was funded by Science Foundation of Health Commission of Heilongjiang Province (2019052), Innovative scientific research funding project of Harbin Medical University (2020-KYYWF-1471), HAI YAN Science Foundation of Harbin Medical University Cancer Hospital (grant no. JJQN2018-18). HAI YAN Science Foundation of Harbin Medical University Cancer Hospital (grant no. JJQN2021-08).

## Conflict of Interest

The authors declare that the research was conducted in the absence of any commercial or financial relationships that could be construed as a potential conflict of interest.

## Publisher’s Note

All claims expressed in this article are solely those of the authors and do not necessarily represent those of their affiliated organizations, or those of the publisher, the editors and the reviewers. Any product that may be evaluated in this article, or claim that may be made by its manufacturer, is not guaranteed or endorsed by the publisher.
