# Supplementary material for: A Comprehensive Evaluation of ZrC Nanoparticle in Combined Photothermal and Radiation Therapy for Treatment of Triple-Negative Breast Cancer
Source: Front Oncol. 2021 Dec 21;11:801352. doi: 10.3389/fonc.2021.801352 (PMC8724783; doi:10.3389/fonc.2021.801352)
Supplement: Supplementary file 1 [file DataSheet_1.pdf]

Supporting Information

A comprehensive evaluation of ZrC nanoparticle in combined photothermal and radiation therapy for treatment of triple-negative breast cancer

Jiang shan[a], Liu zhao[a], Tian yuhang[a], Zhuang ming[b], Piao shiqi[b], Gao yan[c], Andraw Tam[d], Hu hongtao[b*] and Cheng wen[a*] .

[a]Department of ultrasound, Harbin Medical University Cancer Hospital, Nangang District, Harbin, Heilongjiang Province, 150086, P. R. China

[b]Department of radiation oncology, Harbin Medical University Cancer Hospital, Nangang District, Harbin, Heilongjiang Province, 150086, P. R. China

[c]School of Chemistry and Chemical Engineering, Harbin Institute of Technology, Harbin 150001, China

[d]Department of radiation oncology, City of Hope National Medical Center, 1500 East Duarte Road, Duarte CA 91010-3000, United States.

Corresponding author: hrbchengwen@163.com Cheng wen
huht9099@sohu.com Hu hongtao
Jiang shan and Liu zhao contributed equally to this study.

(1)

(2)

(3)

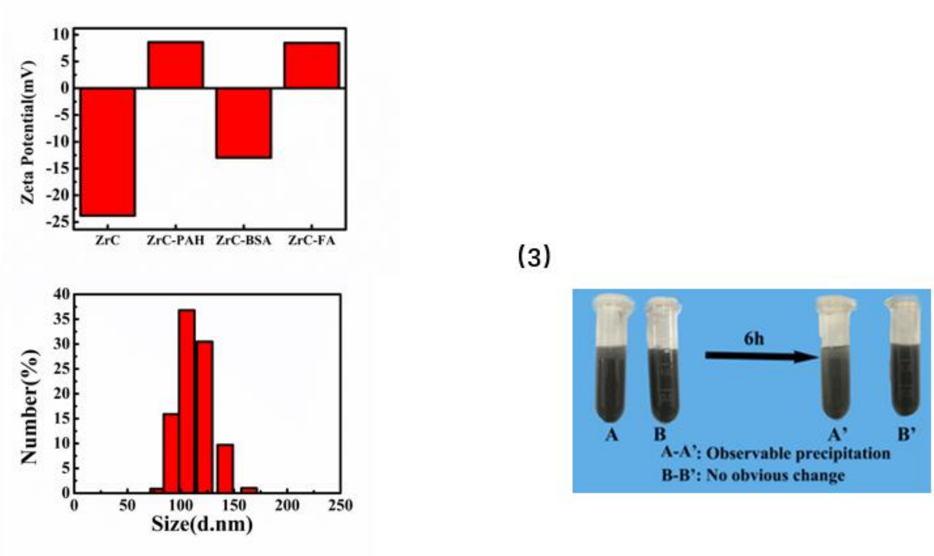

(4)

Z	Element	Family	Atomic Fraction (%)	Atomic Error (%)	Mass Fraction (%)	Mass Error (%)	Fit error (%)
6	C	K	46.55	8.18	10.29	1.18	0.53
40	Zr	K	53.45	11.6	89.71	15.37	0.07

S1: Zeta potentials of as-prepared ZrC NPs,

S2: Dynamic Light Scattering (DLS) data about the size distribution of ZrC NPs

S3: Stability in physiological solutions of ZrC NPs. A is the ZrC powder dissolved in PBS and B is the solution of ZrC NPs.

S4: Analysis of TEM

(5)

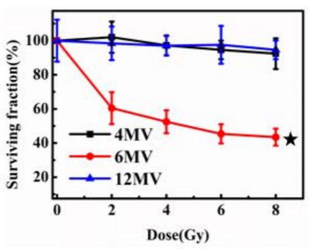

(6)

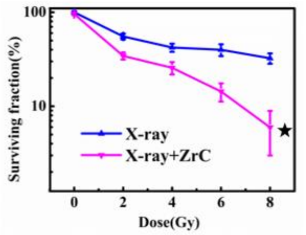

(7)

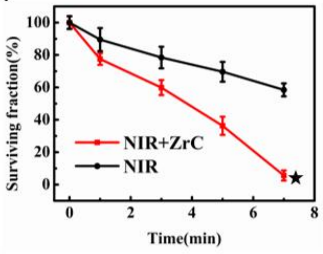

S5, Detection of the appropriate time of NIR irradiation for combine therapy

S6-7, Exploration of effective energy level of X-ray radiation and dose(b-c), Error bars represent mean ± SD. P values based on Student's t-test: *$p < 0.05$.

(8)

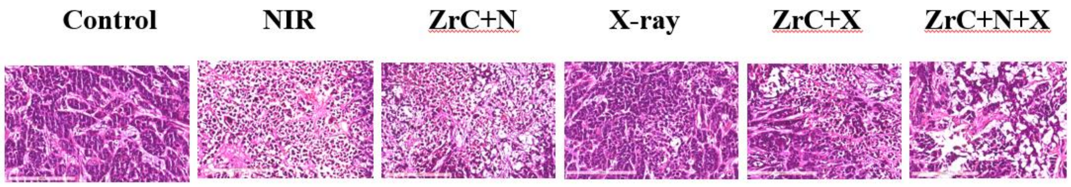

(9)

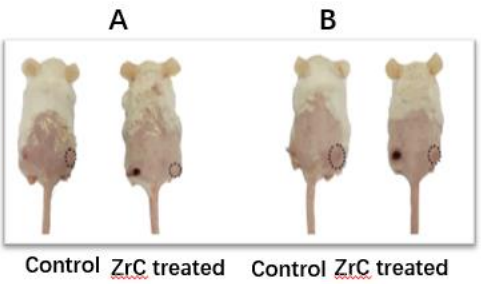

S8: Histology analysis of tumor tissues in (H&E) method.

S9: Pictures of Balb/c mice with bilateral tumors

(10)

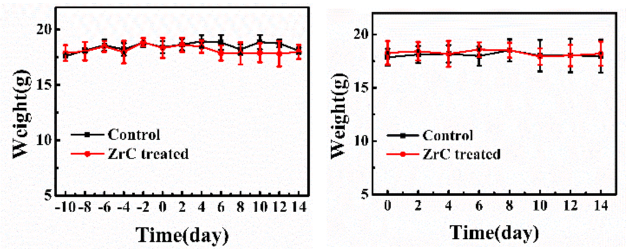

S10: The variations in weight of mice in two groups.

(11)

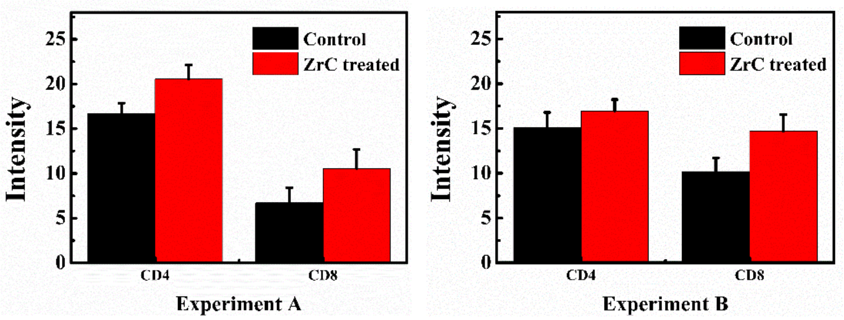

S11, Detect of T lymphocytes in distant tumors in flow cytometry.

(a/b) Error bars represent mean ± SD. P values based on Student's t-test: $*P < 0.05$

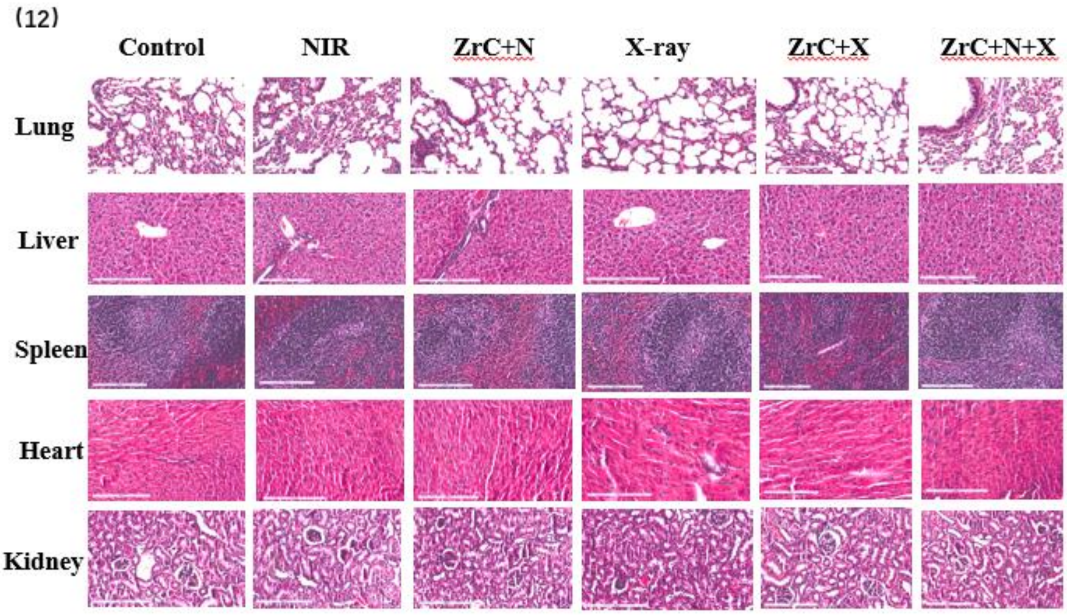

S12

H & E staining image of main organs in healthy female Balb/c mice after treatment.

ZrC+N, ZrC+X and ZrC+N+X groups were treated with intratumoral injection of ZrC NPs (200 µg/mice).

(13)

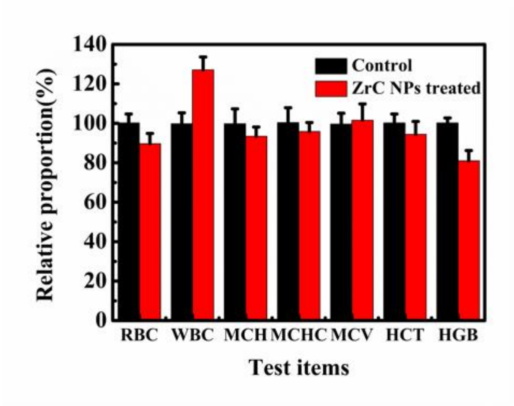

(14)

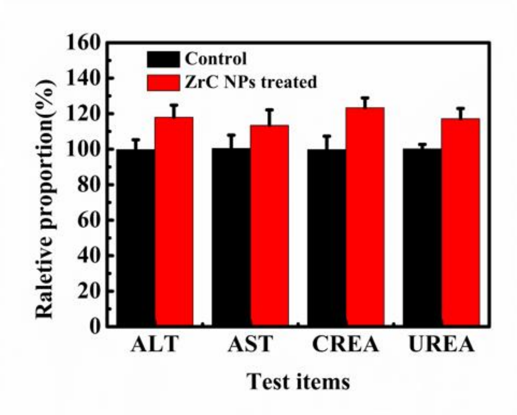

S13: Blood routine analysis of the mice.

S14: Serum biochemical analysis of the mice. Error bars represent mean ± SD.
